# The Divorce Controversy

**Published:** 1920-05-01

**Authors:** 


### THE DIVORCE CONTROVERSY.

The vote in the House of Commons on the divorce law resolution came as a surprise, after the favourable reception which Lord Buckmaster's Bill had received in the Lords. Whether this is to be taken as an instance of the progressive character of the Upper House as compared with the Commons it is not easy to say, for there were many apologists for the Members of Parliament who opposed the resolution, the grounds of opposition being set down to disapproval of some of the conditions of proposed reform, but not all. The whole substance of the debate was entirely on the side of the reformers, and the opponents of the measure who foreshadowed the serious lowering of moral standards were met by facts such as those given by the Secretary for Scotland, who showed that divorce on the ground of desertion had been the law of Scotland since 1573, and none of the fearful consequences now predicted for England in the way of collusion had taken place.

It seems so natural an assumption that hide-bound divorce laws must encourage immorality and involve evil consequences, rather than help in maintaining high standards, that it is difficult to follow out-and-out opposition to reform. Some striking facts were given by Colonel Raw, a medical man and lunacy expert, who told the House that 140,000 persons are certified as insane, and 50,000 are suffering from dementia or degeneration of the brain itself. Between 30,000 and 40,000 of these are married, and their malady is incurable. He also declared that if drunkenness continues for three, four, or five years, there is little hope of cure, and 90 to 95 per cent. of habitual drunkards die drunkards. Is it, therefore, sensible to contend that the marriages of these people must remain indissoluble, save in the case of adultery? Is it humane, is it good for the race, is it fair to the children? The existing laws are harsh and unreasonable, and they make more for loose living and unhappiness than for morality and contentment. What is the value of insisting on the continuance of conditions in which marriage is a mockery and a sham?

The controversy has been enlivened by a bright passage-at-arms between the Lord Chancellor and Canon Lacey in *The Times*. The Canon advised the Lord Chancellor to stick to his legal questions, leaving moral and spiritual questions to others. The Lord Chancellor, in reply, writes with some verve about the arrogance of minor ecclesiastics about proposals by the Canon that are grotesque, and so forth. The next stage in the Parliamentary controversy will be when Lord Buckmaster's Bill goes to the Commons. It continues to receive majority backing in its stages through the Lords, and there is little doubt that it is supported by the general level of opinion in the country.

